# Role of Biomarkers in the Management of Acute Myeloid Leukemia

**DOI:** 10.3390/ijms232314543

**Published:** 2022-11-22

**Authors:** Sara Small, Timothy S. Oh, Leonidas C. Platanias

**Affiliations:** 1 Robert H. Lurie Comprehensive Cancer Center, Northwestern University, Chicago, IL 60611, USA; 2 Division of Hematology-Oncology, Department of Medicine, Feinberg School of Medicine, Northwestern University, Chicago, IL 60611, USA; 3 Division of Hospital Medicine, Department of Medicine, Feinberg School of Medicine, Northwestern University, Chicago, IL 60611, USA; 4 Department of Medicine, Jesse Brown Veterans Affairs Medical Center, Chicago, IL 60612, USA

**Keywords:** biomarkers, acute myeloid leukemia, measurable residual disease, immunotherapy, gene expression analysis

## Abstract

Despite many recent advances in treatment options, acute myeloid leukemia (AML) still has a high mortality rate. One important issue in optimizing outcomes for AML patients lies in the limited ability to predict response to specific therapies, duration of response, and likelihood of relapse. With evolving genetic characterization and improving molecular definitions, the ability to predict outcomes and long-term prognosis is slowly improving. The majority of the currently used prognostic assessments relate to molecular and chromosomal abnormalities, as well as response to initial therapy. These risk categories, however, do not account for a large amount of the variability in AML. Laboratory techniques now utilized in the clinic extend beyond bone marrow morphology and single gene sequencing, to next-generation sequencing of large gene panels and multiparameter flow cytometry, among others. Other technologic advances, such as gene expression analysis, have yet to demonstrate enough predictive and prognostic power to be employed in clinical medicine outside of clinical trials, but may be incorporated into the clinic in the future. In this review, we discuss the utility of current biomarkers, and present novel biomarker techniques and strategies that are in development for AML patients. Measurable residual disease (MRD) is a powerful prognostic tool that is increasingly being incorporated into clinical practice, and there are some exciting emerging biomarker technologies that have the potential to improve prognostic power in AML. As AML continues to be a difficult-to-treat disease with poor outcomes in many subtypes, advances in biomarkers that lead to better treatment decisions are greatly needed.

## 1. Introduction

In a heterogeneous disease such as AML, both predictive and prognostic biomarkers are essential for treatment planning and patient education. Early biomarkers in AML dating back to the 1970s include the French-American-British (FAB) classification system which provided morphology-based differentiation between subtypes of AML that had therapeutic and prognostic implications [1]. The World Health Organization (WHO) further divided AML by recurrent cytogenetic and genetic changes [2,3]. The European Leukemia Network (ELN) classification system (with a newly updated set of recommendations in 2022) also utilizes cytogenetic and molecular biomarkers [4]. As research reveals a deeper understanding of the biology of AML, increasing numbers of biomarkers are being incorporated into these classification schemes and into clinical practice. For example, *FLT3* was the first commonly-mutated gene identified in AML for which a targeted therapy became available [5]. Testing for this mutation is now recommended in all newly-diagnosed AML cases [6]. Here, we summarize the most important biomarkers used clinically at this time, discuss biomarkers that are beginning to be incorporated into clinical practice, and evaluate novel methods that may yield important biomarkers for AML in the future.

## 2. Established Biomarkers in AML

### 2.1. FLT3

Activating mutations of *FLT3* exist in roughly one third of AML diagnoses [7,8]. *FLT3* has a crucial regulatory role in hematopoiesis, and mutations in this gene therefore are important in the pathophysiology of AML [9]. Various *FLT3* mutations have been discovered, including the internal tandem duplication of the JM domain-encoding region (*FLT3-ITD*), representing roughly 25% of AML cases, and mutations around the D835 residue of the TK domain (*FLT3-TKD*), representing approximately 10% of AML cases [8,10]. Previously, AML risk classification related to *FLT3-ITD* was dependent on allelic ratio and *NPM1* mutational status [11], but as of 2022, all *FLT3-ITD* disease is considered intermediate risk [4].

Because of the high frequency and generally poor prognostic significance of *FLT3* mutations, substantial research has focused on the creation of *FLT3* inhibitors for use in frontline, relapsed/refractory, and maintenance settings. The phase III RATIFY study (CALGB 10603), for example, looked at the addition of the tyrosine kinase inhibitor midostaurin to intensive chemotherapy in newly-diagnosed AML with *FLT3* mutations (either *ITD* or *TKD*) and showed an overall survival (OS) benefit with midostaurin compared to placebo (74.7 vs. 25.6 months, respectively) [12]. The phase III ADMIRAL trial compared monotherapy with a newer generation *FLT3* inhibitor, gilteritinib, to salvage chemotherapy in relapsed/refractory AML [13]. The gilteritinib cohort experienced improved rates of CR (34.0%) and OS (median of 9.3 months) compared to the salvage chemotherapy cohort (CR of 15.3%, median OS of 5.6 months) [13].

The phase II German study SORMAIN tested the benefit of adding the tyrosine kinase inhibitor sorafenib as maintenance therapy in adult patients with *FLT3-ITD* AML who achieved a complete response (CR) after allogeneic stem cell transplantation (SCT) and found that sorafenib maintenance reduces disease relapse and death versus placebo by more than half [14]. This trial was also particularly noteworthy as it revealed that measurable residual disease (MRD) status is an important factor in the benefit of sorafenib. In an exploratory analysis, they stratified patients by MRD status pre- and post-transplant. They found that prior to transplant, patients with MRD-negative disease had a significantly better relapse-free survival (RFS) with sorafenib maintenance than placebo maintenance (*p* = 0.028), but there was no significant difference between MRD-positive patients. In contrast, following SCT, MRD-positive patients had an improved RFS with sorafenib maintenance compared to placebo (*p* = 0.015) while MRD-negative patients did not (*p* = 0.191) [14].

### 2.2. IDH1/2

Mutations of isocitrate hydrogenase 1 and 2 (*IDH1* and *IDH2*) genes are found in roughly 8% and 12% of AML cases, respectively, making up one of the most common genetic mutations following *FLT3* [15,16]. Both IDH proteins play important roles in cellular metabolism; mutations in these genes result in the accumulation of 2-hydroxyglutarate, a metabolite that inhibits various cellular regulatory enzymes including histone lysine demethylases [15,17]. As a result, mutations in *IDH1* and *IDH2* lead to higher levels of histone hypermethylation, consequently blocking proper cellular differentiation and maturation, and promoting development of AML [15]. IDH-targeted therapies can release this type of differentiation block, allowing the leukemia cells to undergo maturation and ultimately apoptosis, which also explains the risk for differentiation syndrome as a potential side effect [18].

Use of *IDH1* and *IDH2* as prognostic markers in isolation has been deemed controversial; however, studies have shown the importance of considering the greater genetic and cytogenetic profile in predicting outcomes [16]. For instance, both *IDH* mutations appear to confer worse prognosis in the context of normal cytogenetics with concurrent *NPM1* mutation and in the absence of *FLT3-ITD* mutation [19,20].

Standard treatment for young and healthy patients with either *IDH1* or *IDH2* mutations continues to be induction chemotherapy such as the classic 7 + 3 regimen, followed by consolidative post-remission therapy [15]. In patients who are deemed unfit for intensive induction chemotherapy, the mutant IDH1 inhibitor ivosidenib has been shown to achieve durable remissions as monotherapy in some patients (30.3%) in the frontline setting in a phase I study [21]. Ivosidenib ultimately gained FDA approval as frontline therapy in unfit patients 75 years and older in 2019 [22]. More recent studies have investigated the concurrent use of either ivosidenib or the mutant IDH2 inhibitor enasidenib with intensive induction chemotherapy in the frontline setting [23].

In refractory/relapsed AML, both ivosidenib and enasidenib have shown promise. A phase 1/2 study of ivosidenib in that setting not only led to durable remissions, but also showed promising safety outcomes with low rates of treatment-related adverse events [24]. Enasidenib monotherapy has shown efficacy in patients with relapsed or refractory disease in a phase I/II study [25]. Both agents now have FDA approval for that indication [26,27]. Research to identify mechanisms of resistance to *IDH* inhibitors is also underway [28] and the results of such work may help guide use of such inhibitors in the future.

### 2.3. NPM1

Initially discovered in 2005, *NPM1* mutations are present in roughly 30% of adult AML patients [29]. The NPM1 protein acts as a cytoplasmic shuttling protein and plays important roles in both genomic stability and ribosome synthesis [30,31]. Mutations of the *NPM1* gene leads to abnormal cytoplasmic dislocation of the NPM1 protein, which is thought to contribute to leukemogenesis in AML [29]. *NPM1*-mutated AML is now designated as a distinct entity in the updated 2016 WHO classification system [2]. Based on the updated 2022 ELN guidelines, mutated *NPM1* without *FLT3-ITD* confers a favorable a risk profile. The newer guidelines also outline that *NPM1*-mutated AML with concurrent adverse-risk cytogenetic changes are linked to adverse risk, while research is ongoing regarding the implications of other abnormalities, such as myelodysplasia-related mutations, when paired with mutated *NPM1* [4].

Intensive chemotherapy is the currently recommended therapeutic approach for *NPM1*-mutated AML in young (≤60) and fit individuals [32]. Because *NPM1*-mutated leukemic cells also tend to express the CD33 cell surface antigen, gemtuzumab ozogamicin can be added to standard intensive regimens to improve survival in favorable- and intermediate-risk AML [33]. The favorable impact of the *NPM1* mutation, however, wanes with increasing age [34,35]. Venetoclax-based regimens, such as in combination with hypomethylating agents, have shown promise in older populations [36]. While there are currently no FDA-approved agents with specificity towards mutated *NPM1*, various agents are under investigation, including menin inhibitors, retinoic acid, arsenic trioxide, and dactinomycin [37].

### 2.4. CD33

CD33 is a cell surface antigen that is normally found on immature myeloid precursors and downregulated as part of normal cell maturation [38]. In AML, however, proper cell maturation does not occur, resulting in blast cells that are frequently CD33+ [39], positioning CD33 as a key target for AML therapy [40].

Gemtuzumab ozogamicin (GO) is a CD33-specific antibody-drug conjugate that was introduced as the first targeted therapy for AML in 2000 [41]. The agent is a monoclonal IgG4 antibody connected to a cytotoxic calicheamicin derivative, leading to cell death in CD33+ leukemic blasts [42]. The FDA initially granted accelerated approval for GO in 2000 for CD33+ AML in patients at least 60 years old in their first relapse who were not deemed suitable for conventional cytotoxic chemotherapy [43,44,45,46]. In 2010, however, GO was voluntarily withdrawn from the US New Drug Application by its manufacturer after a follow-up study demonstrated no survival benefit and earlier mortality in newly diagnosed adult AML patients receiving intensive induction chemotherapy in combination with GO [47,48]. Subsequent data on the administration of GO using a fractionated-dosing schedule demonstrated both safety and efficacy, which resulted in its reapproval in combination with intensive chemotherapy in CD33+ AML in adults in the frontline setting in 2017, following the crucial French ALFA-0701 study [33]. GO in combination with chemotherapy has been FDA approved for patients with relapsed/refractory CD33+ AML [49,50].

The decision to add GO to standard intensive chemotherapy is based on risk stratification as per the ELN genetic risk classification system, where GO has been shown to provide a significant survival benefit in cases of favorable-risk AML, and modest benefit for intermediate-risk [51]. Incorporating GO into conventional treatment regimens notably resulted in a 5-year OS improvement of 20% in the core binding factor (CBF) subset of favorable-risk AML [52]. This benefit, however, has not been demonstrated in the adverse risk category [49], and the use of GO in this group is currently not recommended.

### 2.5. TP53

Located on chromosome 17p13, *TP53* codes for a tumor suppressor protein, which is essential for normal cell cycle regulation and response to DNA damage [53]. Mutated *TP53* is present in roughly 5–10% of de novo AML [54] but much more commonly in therapy-related AML and AML with complex karyotype [55]. Within the ELN classification, *TP53*-mutated AML falls under the adverse risk category and confers a remarkably poor prognosis, with a 2-year OS of only 12.8% [4,56]. Not only is *TP53*-mutated AML resistant to standard intensive chemotherapy, but also it has shown resistance to HMAs, such as azacitidine and decitabine, and the BCL-2 inhibitor venetoclax [57]. Rates of relapse are also high in patients who achieve complete remission and proceed to allogeneic SCT [58]. Thus, there is a huge need for more research to develop novel therapeutics for this difficult-to-treat subtype of AML.

Targeted therapies for *TP53*-mutated AML are currently under investigation. Recently, a Phase Ib study investigating the use of magrolimab and azacitidine in frontline mutant *TP53* AML in patients unsuitable for standard intensive chemotherapy has shown promise in achieving durable responses and improving OS [59]. Further investigation is taking place in a phase III trial (ENHANCE-2). Eprenetapopt (APR-246) is a drug that reactivates p53 function [60], restoring p53 function to cells with mutant *TP53*. Combination therapy with azacitidine and APR-246 has demonstrated a synergistic cytotoxic effect and some encouraging preliminary data [60,61,62], but much more data are needed before this regimen can be widely used in this patient group.

### 2.6. ASXL1

Initially reported in myelodysplastic syndrome (MDS), mutations in the *ASXL1* gene have been identified across different myeloid malignancies, including AML [63,64]. *ASXL1* is located on chromosome 20q11 and is involved in the control of gene transcription [65]. Mutations in this gene induce epigenetic dysregulation through abnormal histone modifications, ultimately leading to dysfunctional hematopoiesis and resulting in myeloid malignancies [66]. Mutations of *ASXL1* have been described in approximately 10% of cases of AML [67,68]. Prior studies have described *ASXL1* mutations as an adverse risk factor associated with aggressive disease and resistance to initial chemotherapy leading to worse clinical outcomes [68,69,70]. Recent pre-clinical studies have examined bromodomain and extra-terminal motif (BET) inhibitors as a therapeutic possibility, given that cells harboring mutated *ASXL1* have shown sensitivity to BET inhibitors in mouse models [71]. Further research should help to tailor therapeutics against AML with this gene mutation, given its importance in epigenetic regulation and gene transcription [72].

### 2.7. RUNX1

*RUNX1* has a key function in regulating normal hematopoiesis [73]. *RUNX1* mutations can be divided into germline mutations, responsible for familial platelet disorders and predisposition to AML, and somatic mutations, which occur in both lymphoid and myeloid cancers, including AML [74]. Mutations in *RUNX1* are found in roughly 10% of AML cases and occur in tandem with a complex array of gene mutations, including epigenetic regulators such as *IDH2* and *ASXL1* [75], which confer inferior prognosis [73,76,77]. Research is ongoing to find targeted therapies against mutated *RUNX1*. One promising area of research involves combining protein translation inhibitors, such as omacetaxine, with venetoclax; AML cells with mutated *RUNX1* are more sensitive to this combination than their wild-type counterparts [78]. There is also interest in BET protein antagonists as they restore normal hematopoiesis, cell growth and apoptosis of *RUNX1*-mutated leukemic blast cells, leading to improved survival in xenograft models of AML with mutant *RUNX1* [73].

### 2.8. Cytogenetics

Cytogenetic analysis continues to be very important for the prognosis of patients with AML. The 2017 ELN guidelines outlined specific cytogenetic abnormalities according to their overall risk category [11]. In the case of failure of cytogenetic analysis, FISH analysis can be utilized to identify corresponding gene rearrangements or loss of chromosome material. The favorable risk category includes t(8;21)(q22;q22.1) and inv(16)(p13.1q22) or t(16;16)(p13.1;q22). The intermediate risk category consists of t(9;11)(p21.3;q23.3) and any abnormalities that do not fall under the favorable or adverse risk categories. The adverse risk category consists of t(6;9)(p23;q34.1), t(v;11q23.3), t(9;22)(q34.1;q11.2), inv(3)(q21.3q26.2) or t(3;3)(q21.3;q26.2), and −5 or del(5q); −7; −17/abn(17p). Also included in the high-risk category is complex karyotype, which is defined by three or more cytogenetic aberrations that do not include the aforementioned translocations or inversions, and monosomal karyotype, which requires one monosomy (excluding loss of X or Y) in conjunction with another monosomy or chromosomal abnormality.

The recently published 2022 ELN guidelines include the following additions and modifications [4]: first, *NPM1*-mutated AML in conjunction with poor-risk cytogenetic changes confers adverse risk [79]. Second, the adverse-risk group now includes t(3q26.2;v) and t(8;16)(p11;p13) [80,81]. The updated guidelines now also exclude hyperdiploid karyotypes with multiple trisomies or polysomies from the complex karyotype classification and therefore they no longer confer adverse risk [82]. While there are no FDA-approved therapies that specifically target the vast array of cytogenetic abnormalities in adult AML, the addition of the anti-CD33 antibody-drug conjugate GO to standard chemotherapy appears to provide survival benefit in core binding factor AML, a favorable cytogenetic subgroup that includes t(8;21) and inv(16) [49,52,83].

## 3. Emerging Biomarkers in AML

### Measurable Residual Disease

Measurable residual disease (MRD) refers to the presence of leukemia cells down to levels of one in 10^6^ to one in 10^4^ cells, as compared with one in 100 seen by eye on morphologic assessment. The technologies for measuring MRD, such as quantitative real-time PCR and flow cytometry, have existed for many years, but standardization of the testing methods is ongoing. There is also debate about when and how frequently MRD status should be determined.

Not surprisingly, the presence of residual disease is a negative prognostic indicator in AML, even if the amount of residual disease present is below the threshold for morphologic detection [84]. For example, a study published in 2018 followed 2450 adults with high-risk MDS or AML with standard risk and wild-type *NPM1* who had MRD assessed by multiparameter flow cytometry, and found that outcomes were similar between the patients who had a partial remission and patients who had a CR but remained MRD-positive (5-year OS of 46% versus 51%, respectively) [85]. A meta-analysis of more than 11,000 AML patients reported that the estimated 5-year OS was twice as high for patients that were MRD-negative as compared to those who were MRD-positive (68% vs. 34%, respectively) [86]. The negative prognostic value of MRD also extends to transplant outcomes. For example, one study showed that measurable MRD at any point after transplant predicted relapse within two months [87].

While MRD has clear prognostic importance, it is not necessarily clear how to incorporate it into treatment decisions. The ELN AML MRD expert panel issued an updated series of recommendations for the clinical use of MRD in December of 2021 [88]. These recommendations outline approaches for evaluating MRD by flow cytometry, quantitative real-time PCR, digital PCR (dPCR), and next-generation sequencing, including recommended timing and type of sample. This document also defines positive test results for different scenarios and identifies points at which the detection of MRD may affect further treatment decisions. Importantly, clonal hematopoiesis of indeterminate oncogenic potential (CHIP) mutations do not appear to correlate with relapse rate and therefore are not recommended to be included in MRD assessments [88]. In acute lymphoblastic leukemia (ALL), the bispecific T cell engager blinatumomab is approved for MRD+ disease [89], but at this time there is no FDA-approved treatment approach for AML based on the presence of MRD, and more research is required before drugs or approaches that specifically target MRD+ AML are widely adopted.

## 4. The Future of Biomarkers in AML

### 4.1. Gene Expression Analysis

One promising avenue for AML biomarkers involves the identification of gene signatures by gene expression analysis. For example, one group analyzed 268 AML patients with cytogenetically normal AML obtaining a CR after induction chemotherapy, in hopes of identifying which genes could predict relapse by RNA sequencing (RNA-seq) [90]. They identified a 10-gene signature which includes 7 coding genes and 3 long noncoding RNAs, separate from genes currently used as biomarkers in AML, that were differentially regulated between patients who relapsed and patients who remained in CR for at least 3 years. Their model was able to correctly predict 71%, 86%, and 94% of relapses in the favorable, intermediate, and adverse risk groups, respectively. Another group looked at gene expression levels of *MECOM, ERG, WT1, GATA2, BAALC, MEIS1* and *SPI1* in the bone marrow of 560 newly diagnosed AML patients and found that lower expression of *MECOM* and *MEIS1* correlated with better CR rates, OS, and disease-free survival (DFS) [91].

The technique of single-cell RNA-seq (scRNA-seq) allows for an up-close view of the heterogeneity within the tumor microenvironment or a population of leukemia cells. Van Galen et al. presented a nanowell-based method of analyzing both DNA mutations and transcriptional information from sixteen AML patients and five healthy donors [92]. They were able to use this technology to determine expression signatures and categorize cells by level of differentiation in those patients, which provided more information than DNA sequencing and flow cytometry alone. They also found that the AML cells that promote tumor growth upregulate genes that are key for stress response, redox signaling, proliferation, and self-renewal. When they divided the AML samples into groups based on expression of hematopoietic stem cell (HSC)/progenitor-like genes and granulocyte-macrophage progenitor (GMP)-like genes, the patients with higher HSC/progenitor-like expressions had significantly worse OS as compared to the GMP-like gene signatures, exemplifying the use of single cell gene expression signatures to predict patient outcomes [92].

Stetson et al. used scRNA-seq to analyze differences between matched samples (collected at initial diagnosis and again at relapse) in five different AML patients, and found characteristic RNA changes with AML progression, such as high expression of *CD44*, *HLA*s, and *PTMA* [93].

One limitation of gene expression analyses and scRNA-seq is that transcription profiles are dynamic and may change dramatically depending on the tissue microenvironment, phase of the cell cycle, and in response to different treatments. The ability to monitor these changes in real time would vastly increase the power of this technique and the importance of its incorporation into clinical practice.

### 4.2. Biomarkers for Immunotherapy

Immunotherapy has proved effective in many different tumor types but is still lagging in AML. Potential biomarkers for immunotherapy include receptor expression on tumor or immune cell surfaces, numbers or types of T cells present, and cytokine expression within the tumor microenvironment [94]. The only approved immunotherapy drug in AML to date is GO which targets CD33 on blast cells (discussed above). The ALFA-0701 trial studied the effects of fractionated-dose GO on patients with de novo AML and found the highest efficacy in patients with favorable or intermediate risk disease [33]. Retrospective analyses have yielded contradictory results as to the predictive ability of CD33 positivity on blast cells [95].

Other than the case of GO, immunotherapy has yet to show significant efficacy in AML, though multiple agents are being studied. One of these is flotetuzumab (MGD006), a CD123 × CD3 dual affinity retargeting protein (DART). Based on a phase I/II study of flotetuzumab in patients with relapsed/refractory AML, Uy et al. defined a 10-gene expression signature that predicted response to flotetuzumab [96]. Another group found that *TP53*-mutated disease trended towards better response to flotetuzumab than wild-type *TP53* (ORR of 60% vs. 33.3%, respectively) though this difference did not reach statistical significance [97].

If immunotherapy becomes more commonplace in AML treatment, it will be essential to develop predictive biomarkers to optimally select patients for this type of therapy.

### 4.3. Epigenetics: Methylation Patterns

Epigenetics have long been known to have an important function in AML as well as other cancers. Indeed, dysregulated DNA methylation is characteristic of AML [98], and mutations in the TET protein family (e.g., *TET2*) and DNA methyl transferase proteins (e.g., *DNMT3A*) are common in AML. Hypomethylating agents are now frequently used in AML treatment, without first quantifying global or specific areas of methylation in the genome prior to use.

Experimentally, DNA methylation is readily evaluated by methods such as bisulfite sequencing. One group used The Cancer Genome Atlas to identify methylation of a CpG site at complement component 1 subcomponent R (*C1R)* as a prognostic biomarker for OS in AML patients, related to chromatin organization rather than gene expression levels of *C1R* [99]. Methylation of *C1R* at >27% was associated with a median OS of 53 versus 11 months. Another group found that the methylation status of *ATP11A, ITGAM,* and *ZNRF2* served as accurate prognostic markers in AML patients [100]. Sestakova et al. analyzed previously-touted prognostic DNA methylation markers in AML across fourteen published studies, and confirmed methylation of four genes (*CEBPA, PBX3, LZTS2,* and *NR6A1*) as predictive for longer survival, as well as two other genes (*DLX4* and *GPX3*) [101]. Despite some promising results, methylation patterns of these or other genes are not yet used in clinical practice for AML patients.

### 4.4. Ex Vivo Drug Testing

There are some studies showing the ability of ex vivo drug testing to predict outcomes in patients with AML, as well as multiple commercial products available for ex vivo testing. Thus far, the literature consists mostly of small studies using patient bone marrow samples or peripheral blood to test sensitivity to chemotherapy drugs and targeted agents in tissue culture systems. For example, Lin et al. utilized bone marrow samples from 38 patients with AML to test responses to different AML drugs, by incubating bone marrow cells with various concentrations of drugs, and determining cell viability 72 hours later; they were able to successfully predict clinical treatment outcome in 32/38 cases (84.21%) [102]. Kita et al. found that in thirteen pediatric patients with de novo AML, ex vivo drug sensitivity testing of bone marrow samples correlated with the percentage of MRD and RFS [103]. There are multiple active AML trials utilizing ex vivo drug sensitivity screening listed on clinicaltrials.gov, including one where treatment is chosen based on ex vivo sensitivity to venetoclax (NCT04267081). However, there are many limitations in ex vivo drug sensitivity testing at this time, such a suboptimal representation of the bone marrow niche, lack of drug metabolism in the tissue culture system, and the difficulty of modeling clonal selection over time in short-term in vitro models.

### 4.5. Other Proposed Biomarkers

Another proposed category of biomarkers is proteomic biomarkers. Dowling et al. collected bone marrow samples from AML patients with favorable, intermediate, and unfavorable risk AML, and compared protein expression by mass spectrometry [104]. They found many significant differences between the different groups, such as increased proteins associated with metabolic pathways and biosynthesis of amino acids in the unfavorable group compared to the favorable risk group. Using targeted proteomics analysis, they also found that IL-17A, IL-1RA, IL-1α, and SDF-1α1β were present at significantly different levels in the three AML groups. Kang et al. utilized proteomics to find differentially expressed proteins in extracellular vesicles of AML patients’ bone marrow and then correlated these proteins with survival [105]. Other research groups are combining multiple biomarker methods, such as proteomics and phosphoproteomics and in vitro drug response, to predict AML patients’ response to drugs [106].

There are countless clinical trials underway in AML that incorporate novel biomarkers, evidence of the need for improved biomarkers as well as their importance. A few examples of such biomarkers include the intracellular nucleotide pool as a predictive biomarker for response to induction chemotherapy (NCT03234985), and gene expression in peripheral blood samples on day four after salvage chemotherapy treatment as a predictive marker for treatment response (NCT02527447).

Emerging and future biomarkers are summarized in Table 1.

## 5. Conclusions

Our knowledge of the mutational landscape in AML and implications of these mutations in AML prognosis has expanded significantly in the past few decades. A few of these prognostic biomarkers, such as *FLT3* and *IDH1/2* mutation status, have now become predictive biomarkers with the integration of targeted therapies into common clinical use. However, the complexities in AML extend beyond single gene aberrations, and there is a clear need for additional biomarkers. MRD testing is becoming increasingly sensitive and commonly used, though standardization of these techniques will be important moving forward. As laboratory techniques continue to evolve, there will be an increasing capacity to identify characteristics of disease, and ultimately, it will be up to clinicians to determine the best way to synthesize and utilize this complex information in the management and treatment of AML.

## Figures and Tables

**Table 1:** Emerging biomarkers in AML. qRT-PCR, quantitative real-time PCR; dPCR, digital PCR. NGS, next generation sequencing.

Biomarker	Definition	Examples of Clinical Applications in AML	Clinical Trials
**Measurable residual disease (MRD)**	Presence of leukemia cells on the scale of one in 10^6^ to one in 10^4^ cells. Detectable by flow cytometry, qRT-PCR, dPCR, NGS.	Flow cytometry- and NGS-based assays for determining MRD in first complete remission	NCT05339204 NCT01452646 NCT02870777 NCT03769532
**Gene expression signatures**	Specific genomic alterations and transcriptional profiles identified using RNA sequencing.		NCT00897936 NCT01421862 NCT00897936 NCT01338974 NCT01229956 NCT01057199
**Targets for immunotherapy**	Tumor or immune cell surface receptors and cytokine expression in malignant cells. Identified via single-cell RNA sequencing, mass cytometry, single cell cytokine analysis.	*Monoclonal antibody-drug conjugates* Gemtuzumab ozogamicin (CD33 targeted therapy, FDA-approved) *T-cell directed therapies* AMG 330 (CD33 × CD3 bi-specific T-cell engager) Flotetuzumab (CD123 × CD3 DART) Vibecotamab (CD123 × CD3 DART) CAR-T cell *Checkpoint inhibitors* Nivolumab (PD-L1 inhibitor) Ipilimumab (CTLA-4 blockade) Pembrolizumab (PD-L1 inhibitor) Magrolimab (anti-CD47 checkpoint inhibitor)	NCT02944162 NCT04884984 NCT05023707 NCT04351022
**Epigenetics**	Changes upstream of gene expression. For example, dysregulated DNA methylation patterns identified by methods such as bisulfite sequencing.	Hypomethylating agents (azacitidine, decitabine)	NCT00897936 NCT01421862 NCT00897936 NCT01229956
**Ex vivo drug testing**	Measuring viability of patient samples after treating with various drug combinations in vitro		NCT04267081 NCT03197714 NCT02551718
**Proteomics/** **Phosphoproteomics**	Identification of specific proteins and phosphorylated proteins using non-targeted (e.g., mass spectrometry) and targeted (e.g., multiplex immunoassays) approaches.		NCT01360125 NCT01338974 NCT01057199

## Data Availability

Not applicable.
